# 2-Hydroxypropyl-β-cyclodextrin mitigates pathological changes in a mouse model of retinal cholesterol dyshomeostasis

**DOI:** 10.1016/j.jlr.2022.100323

**Published:** 2022-12-29

**Authors:** Nicole El-Darzi, Natalia Mast, Sandra S. Hammer, Tim F. Dorweiler, Julia V. Busik, Irina A. Pikuleva

**Affiliations:** 1 Department of Ophthalmology and Visual Sciences, Case Western Reserve University, Cleveland, OH, USA; 2 Department of Physiology, Michigan State University, East Lansing, MI, USA

**Keywords:** 2-hydroxypropyl-β-cyclodextrin formulation vehicle, 24-hydroxycholesterol, age-related macular degeneration, Bruch's membrane, cholesterol overload, CYP46A1, diabetic retinopathy, lipid deposition, retina, retinal macrophage activation, AMD, age-related macular degeneration, BLamD, basal laminar deposit, BM, Bruch’s membrane, CC, cholesterol crystal, 27COOH, 5-cholestenoic acid, EC, esterified cholesterol, 24HC, 24-hydroxycholesterol, 27HC, 27-hydroxycholesterol, HFCE, high fat cholesterol-enriched diet, HPCD, 2-hydroxypropyl-β-cyclodextrin, Iba1, ionized calcium-binding adaptor molecule 1, LD, lipid droplet, LXR, liver X receptor, POS, photoreceptor outer segment, RPE, retinal pigment epithelium, SEM, scanning electron microscopy, TC, total cholesterol, TEM, transmission electron microscopy, UC, unesterified cholesterol

## Abstract

CYP46A1 is a CNS-specific enzyme, which eliminates cholesterol from the brain and retina by metabolism to 24-hydroxycholesterol, thus contributing to cholesterol homeostasis in both organs. 2-Hydroxypropyl-β-cyclodextrin (HPCD), a Food and Drug Administration-approved formulation vehicle, is currently being investigated off-label for treatment of various diseases, including retinal diseases. HPCD was shown to lower retinal cholesterol content in mice but had not yet been evaluated for its therapeutic benefits. Herein, we put *Cyp46a1*^*−/−*^ mice on high fat cholesterol-enriched diet from 1 to 14 months of age (control group) and at 12 months of age, started to treat a group of these animals with HPCD until the age of 14 months. We found that as compared with mature and regular chow-fed *Cyp46a1*^*−/−*^ mice, control group had about 6-fold increase in the retinal total cholesterol content, focal cholesterol and lipid deposition in the photoreceptor-Bruch’s membrane region, and retinal macrophage activation. In addition, aged animals had cholesterol crystals at the photoreceptor-retinal pigment epithelium interface and changes in the Bruch’s membrane ultrastructure. HPCD treatment mitigated all these manifestations of retinal cholesterol dyshomeostasis and altered the abundance of six groups of proteins (genetic information transfer, vesicular transport, and cytoskeletal organization, endocytosis and lysosomal processing, unfolded protein removal, lipid homeostasis, and Wnt signaling). Thus, aged *Cyp46a1*^*−/−*^ mice on high fat cholesterol-enriched diet revealed pathological changes secondary to retinal cholesterol overload and supported further studies of HPCD as a potential therapeutic for age-related macular degeneration and diabetic retinopathy associated with retinal cholesterol dyshomeostasis.

The retina is a multilayered neuronal tissue in the posterior part of the eye that absorbs light for subsequent image generation by the brain. Emerging evidence suggest that increased retinal cholesterol levels contribute to the pathogenesis of age-related macular degeneration (AMD) and diabetic retinopathy, two major blinding diseases (1, 2, 3). Cholesterol involvement is particularly evident in AMD as drusen and subretinal drusenoid deposits, the extracellular hallmark lesions of AMD, contain significant amounts (up to 44%) of cholesterol (4, 5, 6). Also, polymorphisms in several cholesterol-related genes have been identified as risk factors for AMD (7).

CYP46A1 is a CNS-specific enzyme, which converts cholesterol to 24-hydroxycholesterol (24HC) (8), a metabolite of cholesterol that can reach the systemic circulation and a potent activator of transcriptional regulation via liver X receptors (LXRs) (9, 10). CYP46A1 plays an important role in retinal cholesterol homeostasis as illustrated by studies of mature 3 to 12 months old *Cyp46a1*^*−/−*^ mice fed cholesterol-free regular rodent chow (contains 5% fat). These animals had almost a 2-fold increase in the levels of retinal cholesterol (11). In addition, both the retina and retinal macrophages/microglia of *Cyp46a1*^*−/−*^ mice showed increased expression of the proinflammatory genes that are normally suppressed when 24HC binds to LXRs. The permeability of the blood vessels in the *Cyp46a1*^*−/−*^ retina was increased as well (11).

2-Hydroxypropyl-β-cyclodextrin (HPCD) is approved by the Food and Drug Administration as a drug delivery vehicle as this cyclic oligosaccharide can encapsulate hydrophobic compounds, thus enhancing their solubility, stability, and bioavailability (12, 13, 14). Depending on the country, HPCD is used in solutions (for oral, intramuscular, or intravenous administration), oral and sublingual tablets, ointments, rectal suppositives, eye drops, and nasal sprays (15, 16). HPCD is known for its ability to lower the levels of tissue cholesterol (17). Hence, HPCD is being tested off-label as a treatment for cholesterol-associated vascular (atherosclerosis and stroke) and neurodegenerative (Niemann-Pick disease type C1, Alzheimer’s, Parkinson’s, and Huntington’s diseases) disorders in both human subjects (18, 19, 20) and animal models (21, 22, 23). The retina has been a target of in vivo cyclodextrin treatments in several studies, which utilized different mouse genotypes, types of cyclodextrins, and administration routes. In *Abca4*^*−/−*^*Rdh8*^*−/−*^ mice, methyl β-cyclodextrin was injected intravitreally and reduced the amount of lipofuscin, a byproduct of the visual cycle (24). In C57BL/6J and *Cyp27a1*^*−/−*^*Cyp46a1*^*−/−*^ mice, HPCD was delivered orally, intraperitoneally, and subcutaneously. This cyclodextrin was shown to cross the blood-retina barriers and reduce the retinal cholesterol levels after only 11 days of treatment, the earliest evaluation time point (25). Finally, in diabetic animals (db/db mice), α-cyclodextrin was injected subcutaneously and reduced the number of cholesterol crystals (CCs) that the retina of these animals was discovered to have (26, 27). Thus, unlike the blood-brain barrier, normally impermeable to HPCD (28), the blood-retinal barrier can be crossed by various cyclodextrins, enabling removal of retinal cholesterol.

In the present work, we continued our investigation of HPCD as a potential pharmaceutical for retinal diseases associated with retinal cholesterol dyshomeostasis (25). We used *Cyp46a1*^*−/−*^ mice, which were put at 1 month of age on a chow high in fat (21% by weight) and enriched with cholesterol (0.2% by weight), that is, resembling a Western diet. In addition, to further increase human relevance, we aged *Cyp46a1*^*−/−*^ mice to 12 months and started HPCD treatment while mice continued to receive high fat cholesterol-enriched (HFCE) diet. Notably, in our assessments of the retinal treatment effects, we focused on those that could provide novel mechanistic insights and could be of therapeutic value. We obtained data suggesting that aged *Cyp46a1*^*−/−*^ mouse on HFCE diet is a suitable model for evaluating therapeutically relevant HPCD treatment effects. Also, this study expanded our understanding of the pathological retinal changes because of retinal cholesterol overload secondary to impaired cholesterol metabolism, diet, and age and demonstrated that HPCD treatment can mitigate these pathological changes.

## Material and Methods

### Animals

Fourteen-month-old female and male *Cyp46a1*^−/−^ mice on the C57BL/6J;129S6/SvEv background were used. These genotype was provided by Dr David Russell (UT Southwestern, Dallas, TX) and generated as described (29). All animals were free of the *Crbl*^*rd8*^ mutation, which was bred out from our colony. Mice were maintained on a standard 12 h light (∼10 lux)-dark cycle with food and water ad libitum. Before weaning, mice were fed regular rodent chow, and after weaning at 1 month of age, they were put on an HFCE diet (TD.88137; Envigo, Indianapolis, IN). All animal experiments were approved by Case Western Reserve University IACUC and conformed to the recommendations of the American Veterinary Association Panel on Euthanasia. Littermates were selected from the pool of all available animals and randomly assigned to either the control group or the treatment group, which were matched by size and sex. Sample size was based on previous experience. The investigators were mostly not blinded with respect to HPCD treatment as they were involved in both animal treatment and subsequent retinal assessments. Only when CCs were imaged in the retina and then counted, investigators were blinded with respect to HPCD treatment. Half of retinal assessments was quantitative and hence could not be affected by investigators’ bias as all data were used and apparent outliers were not excluded. The nonquantitative half of the assessments pertained to histochemistry, immunohistochemistry, and electron microscopy (transmission electron microscopy [TEM] or scanning electron microscopy [SEM]). To minimize investigators’ bias in these assessments, retinal regions that were compared between the control and treatment groups were matched by the retinal location. For SEM, the whole retina was scanned, and equal number of pictures was taken at the same locations in untreated and treated retinas. Images were masked and analyzed in a blinded fashion.

### HPCD administration

Mice were treated with 30% HPCD (catalog no.: 332607; Sigma-Aldrich, St Louis, MO) from 12 to 14 months of age. This solution was prepared by diluting under the hood the 45% HPCD stock in sterile water for injections (catalog no.: 46066-808-25; Aspen Veterinary Resources, Liberty, MO). The dilution was with phosphate-buffered saline sterilized by 0.22 μm filtration (SCGP00525; EMD Millipore Corporation, Billerica, MA). HPCD was usually administered in the morning by subcutaneous injections at a 2 g/kg of body weight dose twice a week for 2 months, the treatment paradigm chosen based on our previous study (25). Control animals received injections with similar amounts of diluted phosphate-buffered saline. No indication of swelling, redness, irritation at the site of injection or of whole-body toxicity was evident for the treatment duration as assessed by lack of mortality or signs of physical distress, such as hunched posture, lethargy, fur ruffling, or respiratory distress. Of pertinence is that a similar HPCD dosing in human subjects with Niemann-Pick disease type C1, except that HPCD was delivered intravenously or intrathecally, raised no safety concerns (20).

### Retinal sterol measurements

Mice were fasted overnight and the following morning were euthanized. Sterols were quantified as described (29, 30) by isotope dilution GC-MS using deuterated sterol analogs as internal standards. The unesterified and/or total sterol content was measured; the latter was the sum of the unesterified and esterified forms of sterols.

### Lipid distribution in the retina

Three dyes were used as described (31, 32). Fluorescent antibiotic filipin (catalog no.: 70440; Cayman Chemical, Ann Arbor, MI) visualized unesterified and esterified (after additional processing) cholesterol (UC and EC, respectively). Fluorescent compound Bodipy 493/503 (D3922; Thermo Fisher Scientific, Inc, Waltham, MA) stained UC, EC, triacylglycerides, and free fatty acids (33). Oil Red O (catalog no.: KTORO; StatLab, McKinney, TX) labeled EC, triacylglycerides, free fatty acids, and retinyl esters (6, 34).

### SEM

Mouse eyes were enucleated and perforated with a 30-gauge needle 1 mm posterior to the limbus. Eyes were placed in freshly prepared 4% paraformaldehyde and kept at room temperature for 8–16 h. Eyes were then transferred to a refrigerator and stored at 4°C. Prior to SEM, eyes were placed in distilled water, lens and vitreous were removed, and eye cup containing retina/retinal pigment epithelium (RPE) was placed in a 24-well dish with distilled water. Retinas were cut with a double-edged razor blade, and the samples obtained were subjected to two different treatment protocols. Samples that we denoted “processed” were fixed for 1 h in 0.1 M sodium phosphate buffer, pH 7.4, containing 1% osmium tetroxide. These samples were next rinsed for 30 min in water and sequentially dehydrated for 15 min in each 25%, 50%, 75%, and 95% aqueous ethanol solutions followed by the three 15 min incubations in 100% ethanol. Samples were critical point dried in a Leica Microsystems model EM CPD300 critical point dryer (Leica Microsystems, Vienna, Austria) using carbon dioxide as the transitional fluid. Samples that we denoted “unprocessed” were obtained after retinas were placed in vapor fixation with 2% osmium tetroxide for at least 48 h. These samples were then mounted on aluminum stubs using high vacuum carbon abs (SPI Supplies, West Chester, PA) and coated with gold (≈30 nm thickness) in an Emscope Sputter Coater model SC 500 (Ashford, Kent, England) purged with argon gas. All samples were analyzed by a JEOL 6610LV (tungsten hairpin emitter) scanning electron microscope (JEOL Ltd, Tokyo, Japan), and the identified crystals were then evaluated for elemental composition using energy-dispersive X-ray spectroscopy (AZtec System, Oxford Instruments, High Wycombe, Bucks, England) (35). Elemental analysis demonstrated mostly carbon and oxygen content, consistent with the chemical structure of cholesterol.

### TEM

This was carried out as described (36) using the osmium-tannic acid-para-phenylenediamine technique to preserve membranes and neutral lipids (37).

### Immunohistochemistry

The preparation of frozen retinal sections and subsequent incubations with rabbit polyclonal anti-Iba1 (ionized calcium-binding adaptor molecule 1) antibody (catalog no.: 019-1974; Wako, Richmond, VA, 1:250 dilution) and Alexa Fluor 647-conjugated goat anti-rabbit IgG (catalog n.: 111-605-144; Jackson ImmunoResearch, West Grove, PA, 1:200 dilution) were as described (29).

### Retinal proteomics

The relative protein abundance in the retina was assessed by the label-free approach as described (38), which was carried out by the Proteomics and Small Molecule Mass Spectrometry Core at Case Western Reserve University (Cleveland, OH). Four biological replicates per group were used, each representing a pooled sample of three retinas from three different female mice. Proteins with nonsignificant changes (*P* ≥ 0.05) in abundance between the treatment and control groups were excluded from the proteomics analysis as are the proteins with less than a 1.2-fold change in their relative abundance even if this change was significant. Protein grouping was based on a biological process as described by UniProt as well as in the literature.

### Data and statistical analysis

Quantitative data were analyzed either by two way-ANOVA with Tukey’s multiple comparison test or a two-tailed unpaired Student’s *t*-test. The sample size (n) is indicated in each figure or figure legend, which also include the breakdown of female and male animals. Statistical significance was defined as ∗*P* ≤ 0.05, ∗∗*P* ≤ 0.01, and ∗∗∗*P* ≤ 0.001. The data and statistical analysis comply with the recommendations on experimental design and analysis in pharmacology (39).

## Results

### Retinal sterol content

**Fig. 1:**
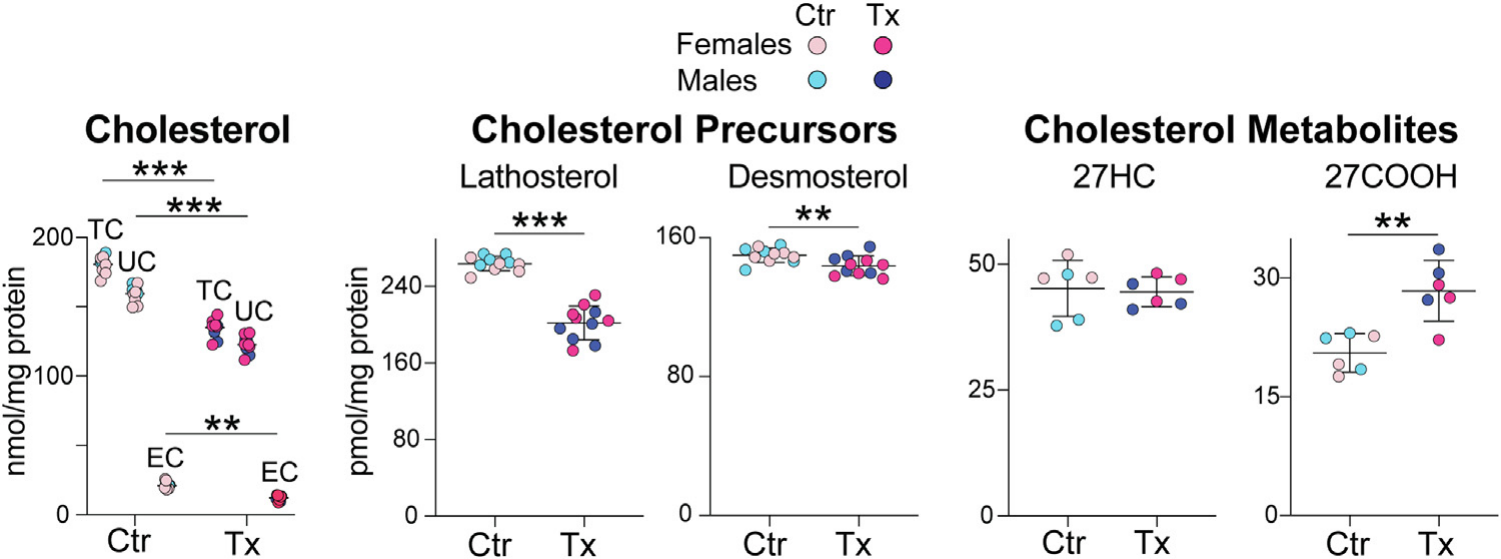
HPCD effect on sterol content in the retina of aged *Cyp46a1*^*−/−*^ mice on HFCE diet. Data represent the mean ± SD of the measurements either in individual retinas (five to six female and five male mice: cholesterol, lathosterol, and desmosterol) or in three samples from female mice and three samples from male mice, each representing combined retinas from three different animals of the same sex (27HC and 27COOH). ∗∗*P* ≤ 0.01; ∗∗∗*P* ≤ 0.001 as assessed by two-way ANOVA with Tukey's multiple comparison test (cholesterol) or a two-tailed unpaired Student’s *t*-test (lathosterol, desmosterol, 27HC, and 27COOH). No statistical significance was found between female (light and dark magenta circles) and male mice (light and dark blue circles) within each group. Ctr, control mice; Tx, HPCD-treated mice.

In control *Cyp46a1*^*−/−*^ mice, representing aged animals on HFCE diet, the content of total cholesterol (TC), UC, and EC was 181, 159, and 22 nmol/mg protein, respectively (Fig. 1). These values were about 6-fold and 5-fold higher than the levels of TC and UC, respectively, as compared with those determined previously in mature (6–12 months old) *Cyp46a1*^*−/−*^ mice on regular rodent chow (TC: 59–64 nmol/mg protein, UC: 24–31 nmol/mg protein, and EC: 28–40 nmol/mg protein) (11). However, in HPCD-treated versus control *Cyp46a1*^*−/−*^ mice, the retinal content was lower for all cholesterol forms: TC (135 vs. 181 nmol/mg protein), UC (123 vs. 159 nmol/mg protein), and EC (12 vs. 22 nmol/mg protein), and represented a -46, -36, and -10 nmol/mg protein change, respectively. The retina of mature (6–12 months old) wild-type mice on regular rodent chow usually contains 34–37, 29–31, and 6 nmol/mg protein of TC, UC, and EC, respectively (11, 40). Accordingly, the absolute values of cholesterol reductions in the HPCD-treated *Cyp46a1*^*−/−*^ retinas were higher than the absolute cholesterol levels in the normal mouse retina. Yet, the relative reductions were smaller (−25%, −23%, and −45% for TC, UC, and EC, respectively) because of the high basal levels of retinal cholesterol.

To gain insight into whether HPCD administration altered retinal cholesterol homeostasis, retinal content of lathosterol, desmosterol, 27-hydroxycholesterol (27HC), and 5-cholestenoic acid (27COOH) was measured (Fig. 1). Lathosterol and desmosterol are the markers of cholesterol biosynthesis in neurons and astrocytes, respectively (41, 42), whereas 27HC and 27COOH are the products of sequential cholesterol C27-hydroxylation by CYP27A1 (43, 44). Like CYP46A1, CYP27A1 eliminates cholesterol from the retina and contributes to the maintenance of the steady-state tissue sterol levels (30, 32). HPCD treatment reduced the lathosterol levels by 62 pmol/mg protein (−23%) and desmosterol levels by 6 pmol/mg protein (−4%). Simultaneously, the treatment increased the 27COOH content by 7 pmol/mg protein (+25%) without affecting the levels of the intermediate product 27HC. Thus, HPCD treatment decreased retinal cholesterol input and increased retinal cholesterol output, that is, induced compensatory (homeostatic) changes, which likely contributed, at least in part, to lowering the levels of retinal cholesterol.

### Retinal distribution of cholesterol

**Fig. 2:**
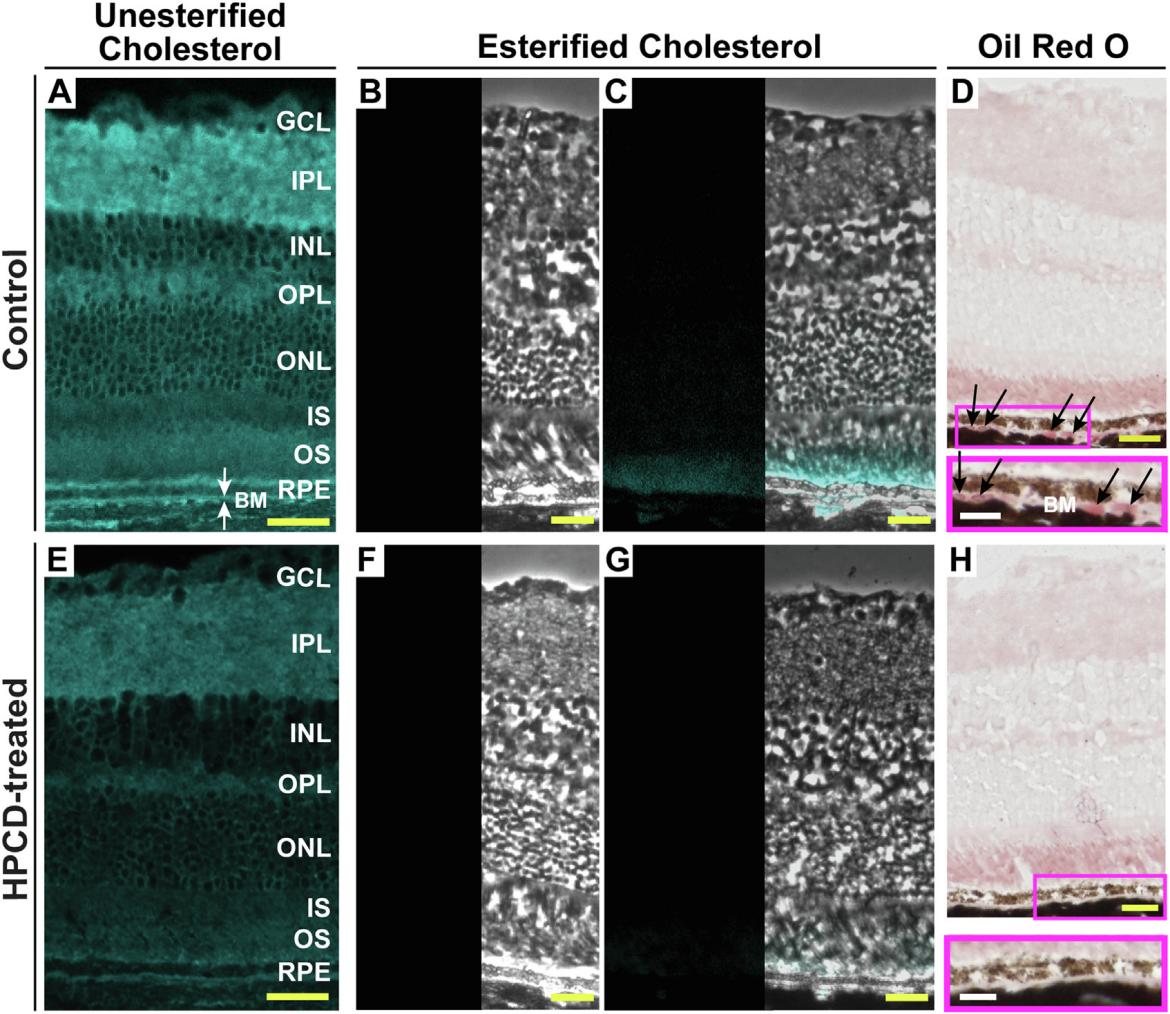
HPCD effect on retinal distribution of cholesterol and Oil Red O-positive lipids. Representative stains (*n* = 5 mice, three females and two males, per group) for UC (A, E); completeness of UC removal (B, F); EC (C, G), and Oil Red O-positive lipids (D, H). B, C, F, and G: Consist of a histochemistry image (on the left) and a phase contrast image overlaid with histochemistry image (on the right). D and H: Magenta rectangles denote the RPE region, which is also shown at a higher magnification. White arrows indicate BM, a five-layer extracellular matrix under the RPE, whose first layer represents the RPE basal lamina (45). Black arrows indicate focal lipid deposits in BM. Yellow scale bars represent 25 μm; white scale bars represent 15 μm. GCL, ganglion cell layer; INL, inner nuclear layer; IPL, inner plexiform layer; IS, inner segment of photoreceptor; ONL, the outer nuclear layer; OPL, outer plexiform layer; OS, outer segment of the photoreceptor.

Both UC and EC were individually stained by filipin. In control *Cyp46a1*^*−/−*^ mice, the filipin fluorescence indicative of UC was bright in all retinal layers and was particularly visible in the apical and basal plasma membranes of the RPE (Fig. 2A). In HPCD-treated mice, retinal fluorescence of UC was not as bright as in control animals and lacked a well-defined signal in the apical and basal sides of the RPE (Fig. 2E). Unlike UC, EC was mainly localized to the photoreceptor outer segments (POS) as well as inside and below the RPE (in Bruch’s membrane [BM], which includes the RPE basal lamina (45)) in control *Cyp46a1*^*−/−*^ mice, yet was barely detectable in those regions in HPCD-treated mice (Fig. 2B, C, F, G). Thus, the HPCD treatment effect was mostly on the cholesterol content in the POS, RPE, and BM. The effect on the latter was particularly important and therapeutically relevant as accumulation of EC and UC in human BM is a major age-related change involved in initiating AMD (3).

### Lipid deposition in BM

Distribution of lipid species other than cholesterol (triacylglycerides, free fatty acids, and retinyl esters) was assessed by retinal labeling with Oil Red O (6, 34). Control and HPCD-treated *Cyp46a1*^*−/−*^ mice had a similar pattern of the retinal Oil Red O staining with the POS being the most intensively stained layer in both groups (Fig. 2D, H). The only difference was that control *Cyp46a1*^*−/−*^ mice had focal lipid deposits in BM, and these deposits were not detectable in BM of HPCD-treated mice. Thus, by using a different lipid stain, we confirmed lipid deposition in BM in control mice and the HPCD effect on this deposition. The participation of the Oil Red O-positive lipids in AMD-specific lesions is now firmly established (6). Hence, the data obtained represent a therapeutically relevant finding.

### Cholesterol crystallization in the retina

**Fig. 3:**
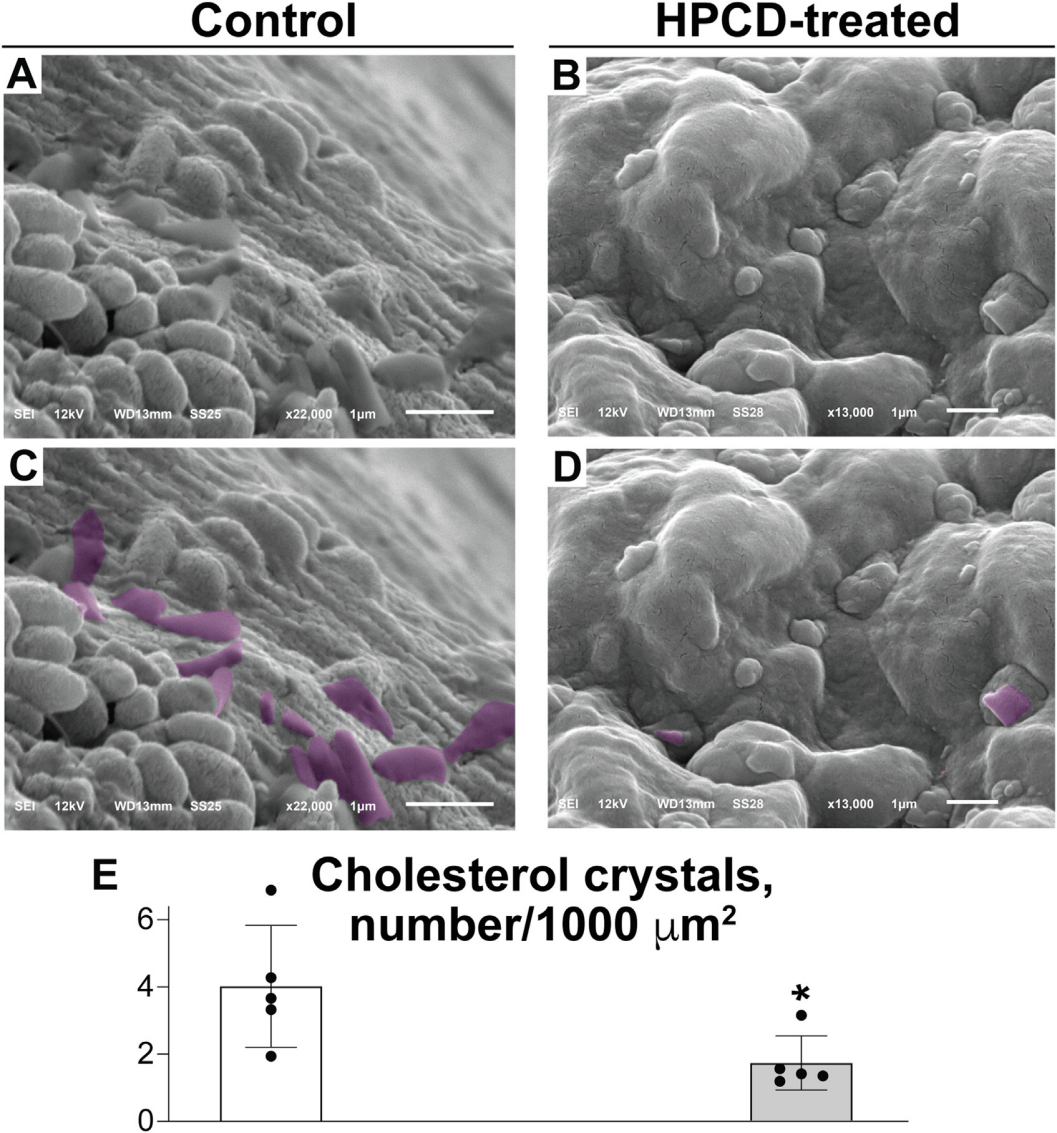
HPCD effect on the number of CCs in the *Cyp46a1*^*−/−*^ retina. SEM images of “unprocessed” retina showing uncolored (A and B) and falsely colored (C and D, violet) CCs in control and HPCD-treated *Cyp46a1*^*−/−*^ retinas. E: The CC quantification. Data represent the mean ± SD of the measurements in individual mice (two female and three male animals per each group) using one to six different images for each animal. Experimenters (six people) who counted crystals were blinded with respect to HPCD treatment. ∗*P* ≤ 0.05 as assessed by a two-tailed unpaired Student’s *t*-test.

A cholesterol-rich environment is known to promote intracellular and extracellular cholesterol crystallization (46, 47), and recently, we discovered that CCs are present in diabetic human and mouse retinas (26). Accordingly, we examined CC formation in the retina of aged *Cyp46a1*^*−/−*^ mice on HFCE diet as this retina is overloaded with cholesterol (Fig. 1). We investigated the interface between the POS and the apical RPE membrane, the region with a high UC and EC content (Fig. 2). This region was also easily accessible by SEM, an imaging approach that enables CC visualization if retinal tissue is not treated with organic solvents (i.e., “unprocessed”), which dissolve CCs (48). In both control and treated groups of mice, CCs were present and had a blade- or plate-like shape (Fig. 3A–D), consistent with the crystal shape formed from cholesterol monohydrate (47). However, the number of CCs per 1,000 μm^2^ was more than two times lower in the HPCD-treated group than in the control group (Fig. 3E), thus demonstrating that a different physical form of cholesterol (i.e., sterol crystal) could be affected by HPCD in addition to the noncrystalline forms of cholesterol. Thus, by using SEM, we demonstrated that retinal cholesterol overload led to the extracellular crystal formation in the control *Cyp46a1*^*−/−*^ retina, and HPCD administration alleviated this process. The reduction in the number of CCs is likely a therapeutically relevant effect of HPCD as in atherosclerosis, CCs are known to represent an insult that exacerbates the disease progression (49, 50). Moreover, CC formation was recently proposed to be a unifying pathogenic mechanism in the development of retinal lesions in diabetic retinopathy (26, 27).

### Retinal macrophage/microglia activation

**Fig. 4:**
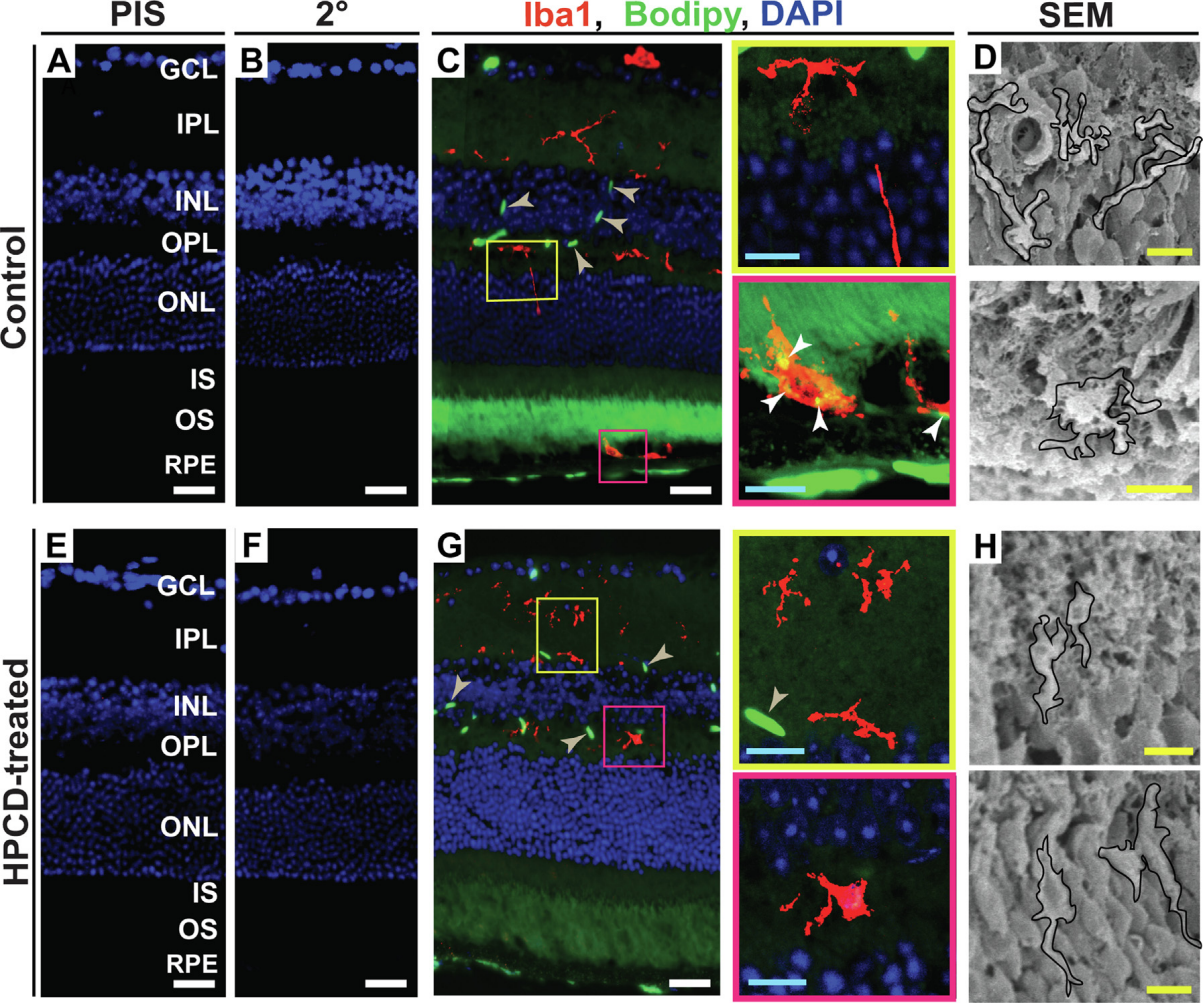
HPCD effect on macrophage/microglia activation in the *Cyp46a1*^*−/−*^ retina. A–C and E–G: Representative images (*n* = 4 mice, three females and one male, per group) of stains for Iba1 (red) and Bodipy (green). Nuclei were stained with DAPI (blue). PIS and 2^o^, control stains using preimmune serum and secondary antibody, respectively. Colored rectangles denote retinal regions shown at a higher magnification. White arrowheads point to colocalization of Iba1 and Bodipy stains; wheat arrowheads indicate Bodipy staining in the retinal blood vessels. D and H: In addition to immunohistochemistry, retinal macrophages/microglia cells (outlined in black) were detected by SEM in the retinal layers corresponding to the colored immunohistochemistry boxes (*n* = 5 mice, two females and three males, per group). White scale bars represent 25 μm; cyan scale bars represent 10 μm; yellow scale bars represent 5 μm. DAPI, 4',6-diamidino-2-phenylindole; GCL, ganglion cell layer; INL, inner nuclear layer; IPL, inner plexiform layer; IS, inner segment of the photoreceptor; ONL, outer nuclear layer; OPL, outer plexiform layer; OS, outer segment of photoreceptor.

Macrophages/microglia integrate lipid metabolism and immune response by engulfing cholesterol excess (or if present, by phagocytizing CCs) and upregulating the proinflammatory gene expression in response to cholesterol overload (50, 51). Macrophages/microglia were found to be activated in the retina of mature *Cyp46a1*^*−/−*^ mice fed regular chow (11). Therefore we investigated the retinal microphages/microglia status in aged *Cyp46a1*^*−/−*^ mice on HFCE diet. Retinal cross sections were first stained for Iba1, a macrophageal/microglial protein specific for both resting and activated cells. Then, sections were incubated with Bodipy (Fig. 4A–C, E–G), a dye, which interacts with multiple lipid species (UC, EC, triacylglycerides, and free fatty acids) (33).

In the retina of control *Cyp46a1*^*−/−*^ mice, the Iba1-positive cells (mostly of amoeboid morphology, i.e., activated) were found in all layers (Fig. 4C), which are normally patrolled by these cells—the nerve fiber layer, the ganglion cell layer, the inner plexiform layer, and the outer plexiform layer (52). In addition, reactive macrophages/microglia cells were detected at the POS-RPE interface (Fig. 4C), where these cell types are typically not found. An independent imaging of the “processed” retinal sections by SEM documented the macrophage/microglia presence at both typical (e.g., outer plexiform layer) and atypical (the POS-RPE interface) retinal locations (Fig. 4D) and thus confirmed our immunohistochemistry findings. Importantly, an overlay of the signals from Iba1 and Bodipy stains (Fig. 4C) revealed cholesterol excess inside macrophages/microglial cells at the POS-RPE interface in the control *Cyp46a1*^*−/−*^ retina, and thereby provided an explanation for the infiltration of the Iba1-positive cells to this region. This was to likely phagocytize cholesterol excess, either in the form of crystals or noncrystalline cholesterol associated with cells, lipoproteins, or lipid droplets (LDs), a known macrophage function (53).

In the retina of HPCD-treated mice, reactive macrophages/microglial cells were also present, yet seemed to have smaller cell bodies (Fig. 4G, H), that is, a rod-like morphology intermediate between the resting and activating phenotypes (54). Yet no macrophages/microglial cells were detected in the subretinal space in this group of mice. Thus, HPCD treatment seemed to reduce both, macrophage/microglia activation and infiltration to the site of retinal insult, the two therapeutically relevant effects, which contribute to the progression of both AMD and diabetic retinopathy (55).

### The RPE-BM region ultrastructure

**Fig. 5:**
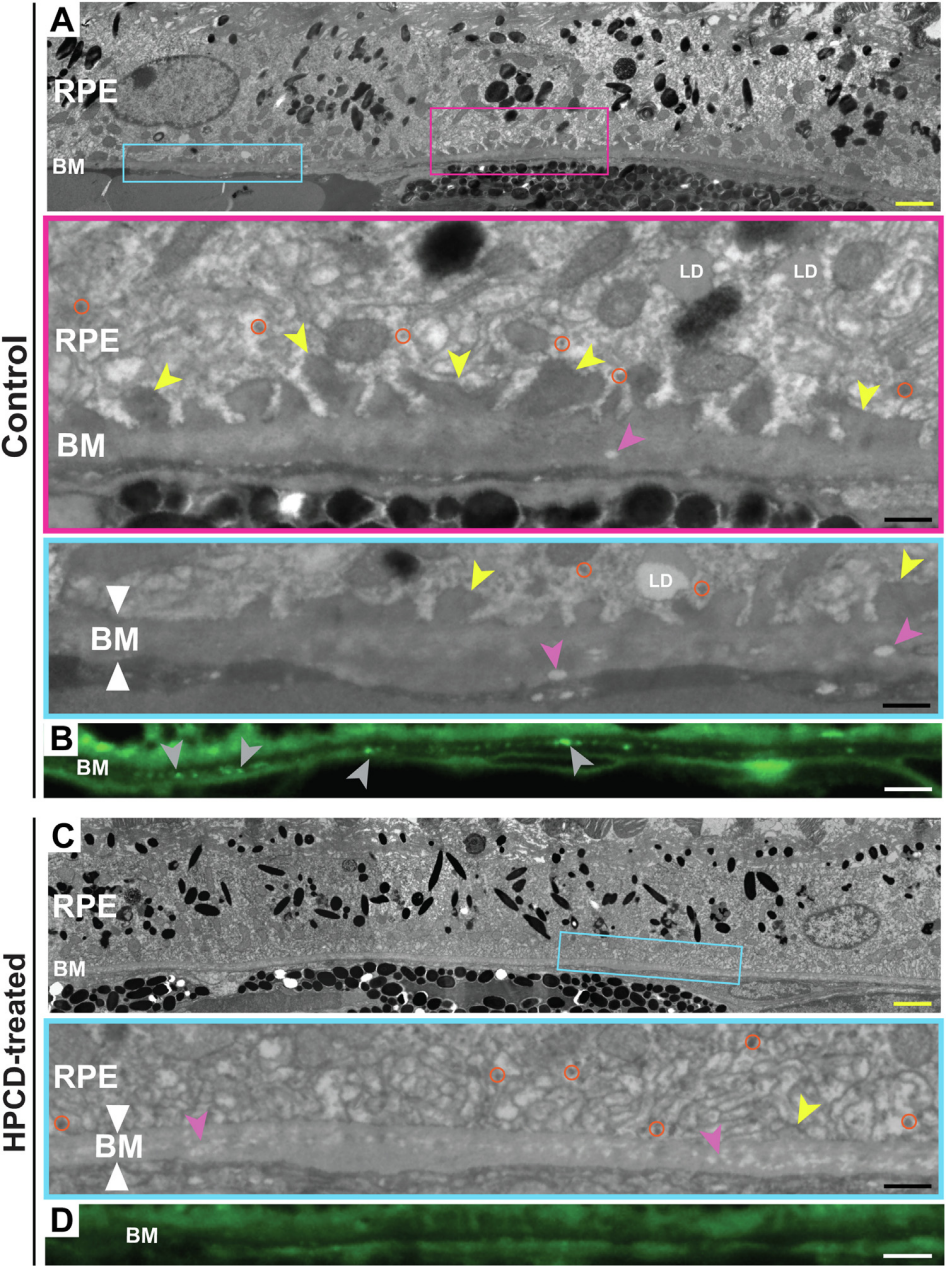
HPCD effect on the RPE-BM region ultrastructure. A and C: Representative images (*n* = 5, three females and two males, per group) of tissue ultrastructure on TEM. Colored rectangles denote the regions shown at a higher magnification. White triangles, borders of BM; yellow arrowheads, basal laminar deposits; magenta arrowheads, electron lucent spaces; orange circles, lipoprotein particles, ∼60 nm in diameter, consistent with the size of an apolipoprotein B-containing lipoprotein particle produced by the RRE (58). B and D: Representative images (*n* = 5, three females and two males, per group) of lipid deposition (gray arrowheads) in BM as assessed by the Bodipy stain. Yellow scale bars represents 2 μm; black scale bars represent 0.5 μm; white scale bars represent 25 μm.

A number of structural changes in the RPE-BM region are associated with pre- or early AMD (3, 56, 57). To ascertain whether they were present in aged *Cyp46a1*^*−/−*^ mice on HFCE diet, we used TEM with the lipid preservation technique as well as Bodipy staining. We found the following abnormalities in control mice. First, the formation of focal basal laminar deposits (BLamDs, Fig. 5A), a consistent finding in AMD, which represents a diffuse and stereotypic thickening of the RPE basement membrane either replacing or incorporating the RPE basal infoldings (56, 57, 59). Second, BM thickening (Fig. 5A), a process, which compromises the metabolic exchange between the choroid and retina, thus affecting photoreceptor function (3). Third, lipid accumulation in BM (Fig. 5B, also shown by Oil Red O staining in Fig. 2D), which eventually forms a lipid wall, a precursor of basal linear deposits and drusen (3). Finally, electron-lucent spaces in BM (Fig. 5A) as these spaces are found in elderly human eyes (60).

However, in HPCD-treated *Cyp46a1*^*−/−*^ mice, BLamDs were not as prominent as in the control group, and their size was much smaller (Fig. 5C). BM was not as thick and electron dense as in control mice (Fig. 5B), in part because of a reduced lipid deposition in BM (Fig. 5D). Finally, electron-lucent spaces in BM were of a smaller size and not as well defined. Thus, HPCD treatment seemed to have some beneficial effects on the BM ultrastructure, which were of relevance to pre- or early AMD.

### Retinal proteomics

**Fig. 6:**
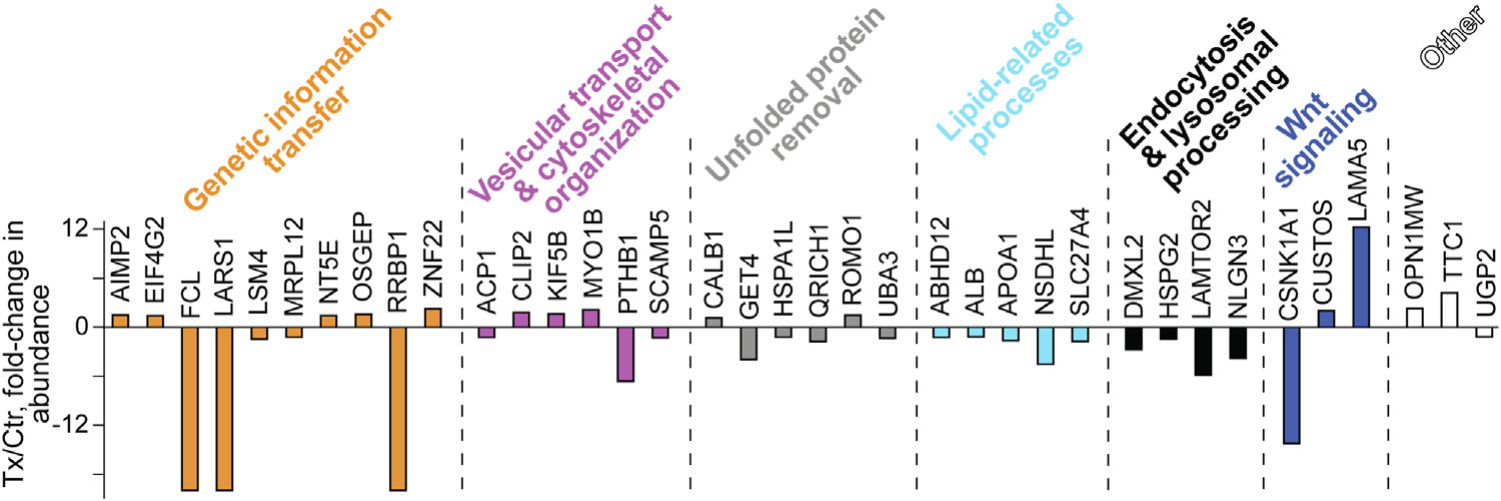
Differentially abundant retinal proteins in HPCD-treated (Tx) versus control (Ctr) *Cyp46a1*^*−/−*^ mice. Protein grouping is by a biological process and shows each protein in only one group despite its involvement in multiple processes.

To gain unbiased mechanistic insights, retinal protein abundance in HPCD-treated versus control *Cyp46a1*^*−/−*^ mice was assessed by the label-free approach. A total of 37 differentially expressed proteins were identified (14 with an increased expression and 23 with a decreased expression), which can be placed in six groups based on a Gene Ontology (http://geneontology.org/) biological process (Fig. 6). Of these groups, two (unfolded protein removal and Wnt signaling) were specific to aged *Cyp46a1*^*−/−*^ mice on HFCE diet and four included the same processes affected previously in HPCD-treated C57BL/6J (wild-type mice) and *Cyp27a1*^*−/−*^*Cyp46a1*^*−/−*^ mice (genetic information transfer, vesicular transport and cytoskeletal organization, lipid homeostasis as well as endocytosis and lysosomal processing (25). The proteins in the genotype-common HPCD-affected processes were different in wild-type, *Cyp27a1*^*−/−*^*Cyp46a1*^*−/−*^, and *Cyp46a1*^*−/−*^ mice with the only exception of DMXL2, which is involved in lysosomal acidification and Ca^2+^-dependent exocytosis (61, 62). DMXL2 had a common change (a decrease) in abundance in all three HPCD-treated genotypes (25). The abundance of all other proteins in the endocytosis and lipid-related groups was decreased in HPCD-treated *Cyp46a1*^*−/−*^ mice as well, including NSDHL and APOA1 participating in cholesterol biosynthesis (63) and intraretinal cholesterol transport (64), respectively (Fig. 6). Changes in these proteins were consistent with decreases in retinal cholesterol biosynthesis (NSDHL) and retinal cholesterol overload (APOA1) detected by the sterol quantifications (Fig. 1). Thus, the retinal proteomics analysis documented *Cyp46a1*^*−/−*^-specific and genotype-common biological processes affected by HPCD treatment and identified some of the proteins, which could mediate the HPCD effects in *Cyp46a1*^*−/−*^ mice.

## Discussion

The present work is a continuation of our previous studies of *Cyp46a1*^*−/−*^ mice (11) and HPCD, which was shown to cross the blood-retinal barriers in mice, when administered intraperitoneally, orally, or subcutaneously (25). Normally, CYP46A1 is only expressed in neurons of the brain and retina and in addition, in the RPE (65, 66, 67). Hence, *Cyp46a1*^*−/−*^ mice, which cannot convert cholesterol to 24HC, represent a model of the CNS-specific impairment of cholesterol removal. We found that even on a regular cholesterol-free diet, mature *Cyp46a1*^*−/−*^ mice versus wild-type animals had a retinal cholesterol overload (59–64 nmol/mg protein vs. 34–37 nmol/mg protein) as well as an upregulation of *Ccl2*, *Cox-2*, *Cxcl1*, *iNos*, *Il-1β*, *Il-6*, and *Tnfα*, that is, a set of the proinflammatory genes, which is usually transrepressed by 24HC activation of LXRs (11). Nevertheless, retinal function in *Cyp46a1*^*−/−*^ mice on normal diet remained unchanged as indicated by their electroretinography responses (11).

Our previous investigation of HPCD was as a proof-of-concept demonstration that HPCD treatment can reduce retinal cholesterol levels in mice when the sterol levels are normal (C57BL/6J mice) or increased by genetic ablation of the two cholesterol-related genes (*Cyp27a1*^*−/−*^*Cyp46a1*^*−/−*^ mice) (25).

In the current work, we put *Cyp46a1*^*−/−*^ mice on a chow with the elements of a western diet (high fat and enriched with cholesterol) and aged them until 12 months to further exacerbate their retinal phenotype. The retinal levels of TC in control *Cyp46a1*^*−/−*^ mice almost tripled, that is, became 181 nmol/mg protein (Fig. 1), and were the highest of any other mouse genotype we ever characterized (e.g., C57BL/6J, *Cyp27a1*^*−/−*^*, Cyp46a1*^*−/−*^, *Cyp27a1*^*−/−*^*Cyp46a1*^*−/−*^, *ApoE*^*−/−*^, *ApoD*^*−/−*^, *ApoD*^*−/−*^*ApoE*^*−/−*^, *Soat1*^*−/−*^, *Cyp27a1*^*−/−*^*Cyp46a1*^*−/−*^*Soat1*^*−/−*^, and *Cyp27a1*^*−/−*^*Cyp46a1*^*−/−*^*Apoe*^*−/−*^ mice (11, 29, 36, 38, 40). Importantly, the cholesterol excess in the control *Cyp46a1*^*−/−*^ retina was mainly accumulated in the POS, RPE, BM as well as the POS-RPE and RPE-BM interfaces (Fig. 2, Fig. 3, Fig. 4, Fig. 5); the latter two being the pathophysiologically relevant locations for subretinal drusenoid deposit and soft drusen, respectively (4, 6). Thus, aged *Cyp46a1*^*−/−*^ mice on HFCE diet enabled us to monitor the HPCD effects on the extracellular lesions (EC/lipid and/or CC accumulation) on both sides of the RPE, which was an advantage as compared with available mouse models that exhibit EC accumulation only in BM (68, 69, 70).

HPCD is suggested to lower tissue cholesterol via several mechanisms. These include cholesterol extraction from the plasma membranes and either retention within HPCD or transfer to circulating apolipoprotein particles (71). HPCD can also enter cells via endocytosis/pinocytosis and reach the late endosomal/lysosomal compartment (25, 72, 73). HPCD can then be released from the lysosomes with or without lysosomal cholesterol and leave cells via stimulation of the lysosomal exocytosis (25, 74). In addition, HPCD can stimulate the lysosomal cholesterol release into the cytoplasm for subsequent cellular processing (75, 76, 77). This processing can include cholesterol esterification by SOAT1 or hydroxylation by tissue- and organelle-specific cholesterol-metabolizing enzymes CYP46A1, CYP27A1, CYP11A1, and CYP7A1.

**Fig. 7:**
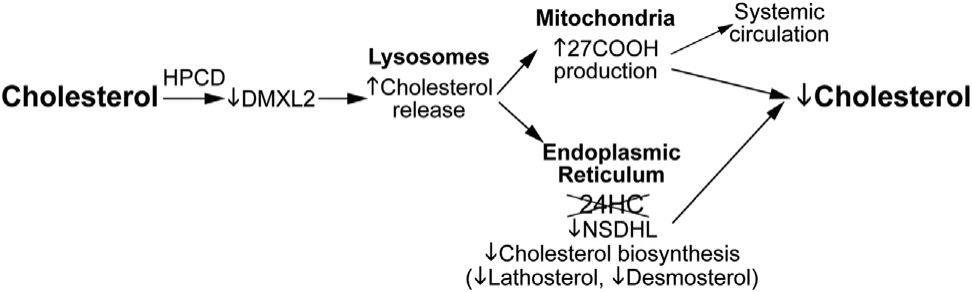
Proposed model of HPCD-induced intracellular cholesterol reduction in the *Cyp46a1*^*−/−*^ retina. A decrease in the DMXL2 abundance could affect lysosomal cholesterol processing and leads to an increase in the lysosomal cholesterol release. This additional cholesterol could then traffic (possibly by vesicular transport) to both mitochondria and endoplasmic reticulum for metabolism to oxysterols. However, in the endoplasmic reticulum of *Cyp46a1*^*−/−*^ mice, cholesterol excess can no longer be hydroxylated to 24HC by CYP46A1. Hence, there could be a transient accumulation of the microsomal cholesterol in the retina of HPCD-treated *Cyp46a1*^*−/−*^ mice, and as a result, a compensatory NSDHL downregulation and a reduction in retinal cholesterol biosynthesis as indicated by decreases in the levels of retinal lathosterol and desmosterol. However, in the retinal mitochondria of HPCD-treated *Cyp46a1*^*−/−*^ mice, this additional lysosomal cholesterol could be efficiently metabolized by CYP27A1 and converted to 27HC and then to 27COOH, which can quickly reach the systemic circulation. Additional mechanisms could also be operative as abundance of proteins in other functional groups was affected by HPCD treatment as well. Yet functional significance of these changes remains to be determined.

Our assessments provided novel mechanistic insights into HPCD effects both inside and outside retinal cells. Indeed, a combination of retinal sterol quantifications and retinal proteomics pointed to the potential intracellular HPCD mechanisms. Specifically, in *Cyp46a1*^*−/−*^ mice, HPCD treatment reduced the levels of retinal TC by 25%, UC by 23%, and EC by 45% (Fig. 1) and simultaneously increased the levels of retinal 27COOH by 25% (Fig. 1). 27COOH is a product of a mitochondrial enzyme CYP27A1, which unlike cholesterol can rapidly diffuse out of cells (78). In addition, there were decreases in abundance of DMXL2 (2.8-fold, Fig. 6), whose loss was shown to affect endolysosomal homeostasis because of a decrease in the lysosomal pH (79) as well as of NSDHL (4.5-fold), a microsomal cholesterologenic enzyme, whose transcription is suppressed by cellular cholesterol excess (80). Based on these findings, we propose a multistep mechanism that could underlie retinal intracellular cholesterol reduction in HPCD-treated *Cyp46a1*^*−/−*^ mice (Fig. 7).

Filipin histochemistry, TEM, SEM, and Iba1 immunohistochemistry provided mechanistic insights regarding both intracellular and extracellular HPCD effects. Specifically, filipin histochemistry suggested that HPCD treatment lowered the accumulation of EC in the POS-BM region (Fig. 2). EC is an inert form of cholesterol, generated to protect cells from the toxicity of UC excess (81). EC and UC undergo a continued cycle of de-esterification-esterification to maintain cholesterol homeostasis, and β-cyclodextrins were reported to sequester not only UC but EC as well (82, 83). Hence, HPCD treatment could reduce retinal EC in *Cyp46a1*^*−/−*^ mice by interacting with UC and thus shifting the equilibrium toward cholesterol de-esterification. Alternatively, if like other cyclodextrins (84, 85), HPCD could directly interact with EC, this could also be a mechanism for a reduction of the EC content.

EC is usually stored in LDs comprised of a neutral lipid core (mainly EC along with triglycerides and retinyl esters) surrounded by a monolayer of amphipathic lipids (phospholipids and UC) and LD-associated proteins (86). When the concentration of UC within the LD membrane exceeds the saturation threshold or the ability of phospholipid head groups to cover all UC molecules, UC precipitates adjacent to the membrane, forming the crystals of cholesterol monohydrate (86). The latter could be a mechanism for the formation of CCs in control *Cyp46a1*^*−/−*^ mice on HFCE diet as the accumulation of EC was the highest in the POS (Fig. 2), and CCs were detected at the POS-RPE interface (Fig. 3). Accordingly, a reduction of the CC load as a result of HPCD treatment (Fig. 3) could either be due to a general reduction in the levels of EC and UC or a direct HPCD interaction with CCs, thus leading to their dissolution as shown previously (21).

CCs are avidly phagocytized by macrophages (53), consistent with the Iba1 immunohistochemistry and Bodipy stain, which documented cholesterol excess in the macrophage/microglia at the POS-PRE interface in control *Cyp46a1*^*−/−*^ mice (Fig. 4). However, CCs are firm and sharp and therefore can physically damage the plasma membranes of phagocytosing macrophages. In addition, CCs can activate the macrophageal NLRP3 inflammasome and induce macrophageal apoptosis (47, 50, 53). Hence, the CC phagocytosis can lead to macrophage activation, in agreement with the cell shape on the Iba1 immunostaining (Fig. 4). In addition, CCs are an established endogenous danger signal and can elicit the innate immune response by activating the lectin, classical, and alternative complement pathways with subsequent release of proinflammatory cytokines like TNFα and IL-1β (87, 88).

The macrophage/microglia activation was reduced in HPCP-treated *Cyp46a1*^*−/−*^ mice (Fig. 4), possibly as a result of reductions in the retinal UC and EC levels (Fig. 1) and/or direct interaction of HPCD with CCs. The latter occurs in human plasma and suppresses the CC-induced inflammation. HPCD-bound CCs were shown to elicit a reduced production of reactive oxygen species and proinflammatory cytokine responses and had a lesser deposition on them of IgGs, pattern recognition molecules, and complement factors with subsequent lesser complement activation (89). Regardless of the mechanism, the HPCD-induced reduction in the macrophage/microglia activation is an important finding as complement and macrophage/microglia activation are suggested to be important contributors to AMD pathogenesis (90, 91). Similarly, the role of inflammation, initiated by immune cell activation and production of inflammatory molecules, is increasingly more appreciated as a mechanism for progression of diabetic retinopathy (92, 93).

Finally, TEM, along with Oil Red O and Bodipy staining, showed improvements after HPCD treatment in the ultrastructure of the RPE-BM region (Fig. 5), which included a reduction in BLamD, BM thinning, and decreased lipid deposition in BM. These beneficial effects could be secondary to a decreased cholesterol and lipid load in this region, although other mechanisms (e.g., changes in protein expression because of HPCD treatment) cannot be excluded. Future studies are necessary to ascertain this ultrastructural HPCD effect. In the future, we will also evaluate whether there is a change in retinal function in our model and if so, whether HPCD treatment improves retinal function.

In conclusion, aged *Cyp46a1*^*−/−*^ mice on HFCE diet were used as a model of retinal cholesterol dyshomeostasis and cholesterol accumulations on both sides of the RPE. This model received subcutaneous injections of HPCD, which elicited multiple and therapeutically relevant effects. The observed effects included homeostatic responses leading to a reduction of the overall retinal cholesterol excess as well as decreases in focal cholesterol depositions in the pathophysiologically relevant AMD and diabetic retinopathy locations. Our animal model was discovered to have CCs at the POS-RPE interface, an important finding that enhances our understanding of the pathological mechanisms leading to retinal damage. The CC number was decreased after HPCD treatment leading to a reduction in retinal microphage/microglia activation and infiltration of these cells to the POS-RPE interface. Overall, this study supports further investigation of HPCD as a therapeutic agent for retinal diseases associated with retinal cholesterol dyshomeostasis.

## Data availability

The data that support the findings of this study are contained within the article.

## Conflict of interest

The authors declare that they have no conflicts of interest with the contents of this article.
